# Supplementary material for: The interactome of intact mitochondria by cross-linking mass spectrometry provides evidence for coexisting respiratory supercomplexes
Source: Mol Cell Proteomics. 2017 Dec 8;17(2):216–32. doi: 10.1074/mcp.RA117.000470 (PMC5795388; doi:10.1074/mcp.RA117.000470)
Supplement: Supplemental Data [file supp_RA117.000470_133922_0_supp_23978_fzffwf.zip › spectra_annotation/mito_DR_spectra_annotation/374-1-4-1-3-1.pdf]

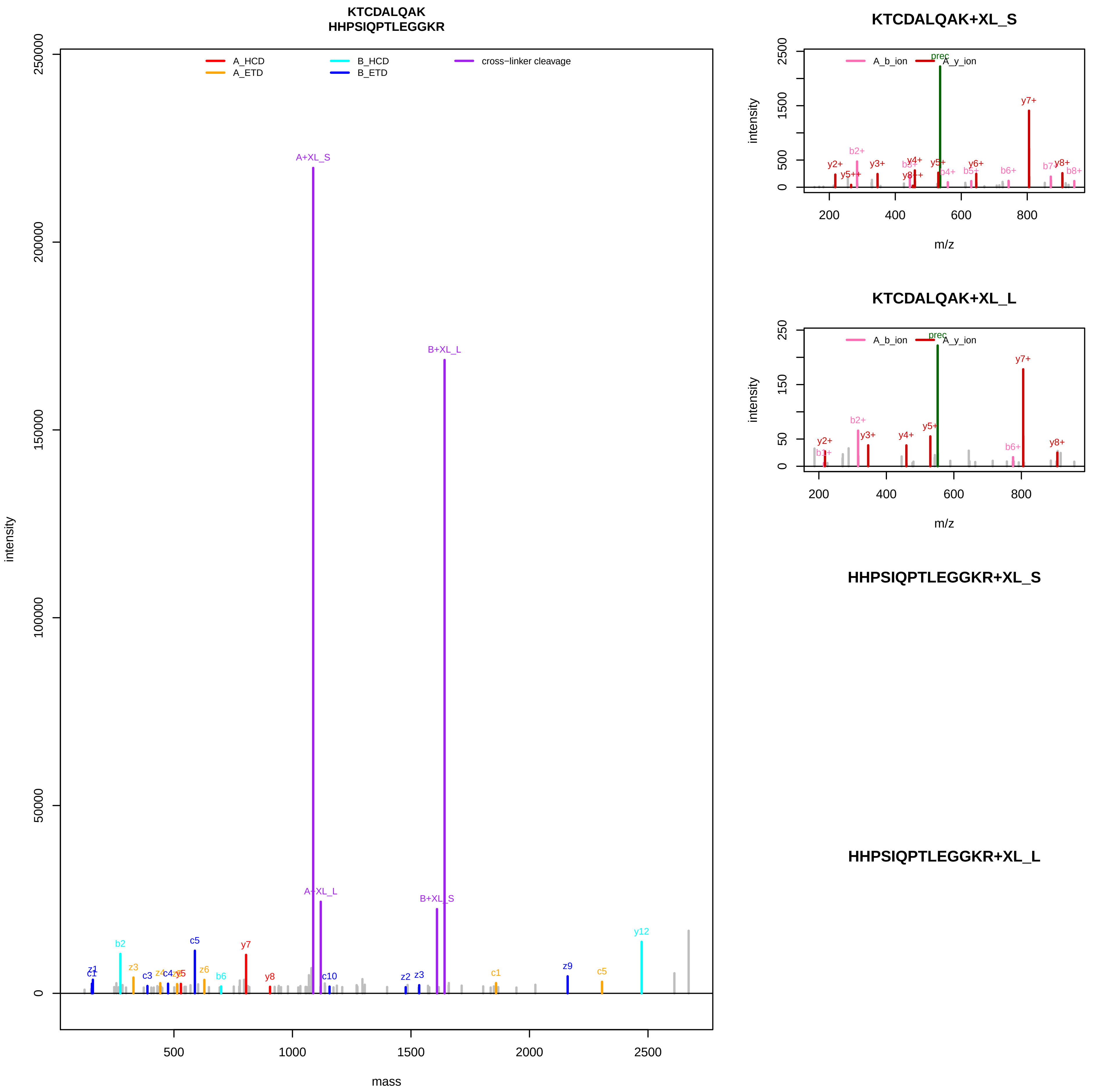

KTCDALQAK
HHPSIQPTLEGGKR
A_HCD
A_ETD
B_HCD
B_ETD
cross−linker cleavage
intensity
mass
0
50000
100000
150000
200000
250000
500
1000
1500
2000
2500
A+XL_S
B+XL_L
A+XL_L
B+XL_S
z1
c1
b2
z3
c3
z4
c4
y5
z6
b6
c5
y7
y8
c10
z2
z3
c1
z9
c5
y12
KTCDALQAK+XL_S
A_b_ion
A_y_ion
prec
y7+
b2+
y2+
y3+
y5++
y4+
y5+
y6+
b6+
b7
y8+
b8+
b4+
b5+
y8++
m/z
0
500
1500
2500
200
400
600
800
KTCDALQAK+XL_L
A_b_ion
A_y_ion
prec
y7+
b2+
y2+
y3+
b3+
y4+
y5+
b6+
y8+
m/z
0
50
150
250
200
400
600
800
HHPSIQPTLEGGKR+XL_S
HHPSIQPTLEGGKR+XL_L
